# A visual quality control scale for clinical arterial spin labeling images

**DOI:** 10.1186/s41747-018-0073-2

**Published:** 2018-12-19

**Authors:** S. M. Fallatah, F. B. Pizzini, B. Gomez-Anson, J. Magerkurth, E. De Vita, S. Bisdas, H. R. Jäger, H. J. M. M. Mutsaerts, X. Golay

**Affiliations:** 1 Department of Brain Repair and Rehabilitation, UCL Institute of Neurology, London, UK; 2 The National Hospital for Neurology and Neurosurgery, London, UK; 3 Radiology Department, King Abdualaziz Medical City, Riyadh, Saudi Arabia; 4 Neuroradiology, University Hospital of Verona, Piazzale Stefani 1, 37126 Verona, Italy; 5 Unitat Neuroradiologia, Hospital de la Santa Creu i Sant Pau, IIB-Sant Pau, Universitat Autonoma, Barcelona, Spain; 6 Radiology Department, Academic Medical Center, Amsterdam, The Netherlands; 7 Radiology Department, Vrije Universiteit University Medical Center, Amsterdam, The Netherlands; 8 Radiology Department, University Medical Center Utrecht, Utrecht, The Netherlands

**Keywords:** Arterial spin labelling, Magnetic resonance imaging, Perfusion imaging, Quality control

## Abstract

**Background:**

Image-quality assessment is a fundamental step before clinical evaluation of magnetic resonance images. The aim of this study was to introduce a visual scoring system that provides a quality control standard for arterial spin labeling (ASL) and that can be applied to cerebral blood flow (CBF) maps, as well as to ancillary ASL images.

**Methods:**

The proposed image quality control (QC) system had two components: (1) contrast-based QC (cQC), describing the visual contrast between anatomical structures; and (2) artifact-based QC (aQC), evaluating image quality of the CBF map for the presence of common types of artifacts. Three raters evaluated cQC and aQC for 158 quantitative signal targeting with alternating radiofrequency labelling of arterial regions (QUASAR) ASL scans (CBF, T1 relaxation rate, arterial blood volume, and arterial transient time). Spearman correlation coefficient (*r*), intraclass correlation coefficients (ICC), and receiver operating characteristic analysis were used.

**Results:**

Intra/inter-rater agreement ranged from moderate to excellent; inter-rater ICC was 0.72 for cQC, 0.60 for aQC, and 0.74 for the combined QC (cQC + aQC). Intra-rater ICC was 0.90 for cQC; 0.80 for aQC, and 0.90 for the combined QC. Strong correlations were found between aQC and CBF maps quality (*r* = 0.75), and between aQC and cQC (*r* = 0.70). A QC score of 18 was optimal to discriminate between high and low quality clinical scans.

**Conclusions:**

The proposed QC system provided high reproducibility and a reliable threshold for discarding low quality scans. Future research should compare this visual QC system with an automatic QC system.

## Key points

- ASL quality control guidelines and standards of acceptance are needed for clinicians
- Visual quality control score is able to select clinically useful scans
- This quality control shows reasonable reproducibility and reliability
- Quality control can be applied to various ASL sequences

## Background

Arterial spin labeling (ASL) is a non-invasive magnetic resonance imaging technique that uses magnetically labelled blood water as an endogenous diffusible tracer to quantify cerebral blood flow (CBF) [1]. Because of the tight coupling between brain perfusion and neuronal health, ASL has shown to be an indispensable tool to study brain function *in vivo* [2–4]. Its non-invasiveness and the lack of an injectable tracer allows longitudinal monitoring of disease progression and treatment efficacy [5].

For the translation of ASL to clinical practice, a wide range of significant developments were performed [6]. Image quality has been improved [7], acquisition times have been reduced [8] and the reliability and reproducibility of ASL perfusion images has been established for multiple centres with different scanners and sequences [9, 10]. Standardised acquisition methods were agreed upon [1], physiological perfusion confounders were reviewed [11] and standardised image processing methods are developed [12, 13]. One lacking step for enabling translation of ASL to clinical practice and clinical trials is the development and validation of standardised quality control (QC) guidelines [1].

Typically, ASL provides CBF as a single measure of perfusion. However, ASL techniques can be modified to acquire CBF images at multiple post-labelling delays. This offers more information about the labelled bolus and its arrival to the tissue, providing more comprehensive haemodynamic parameters [14, 15]. One of these techniques is the quantitative signal targeting with alternating radiofrequency labelling of arterial regions (QUASAR) [16]. In addition to CBF maps, QUASAR acquires several other ancillary parametric maps in the same resolution and space as the ASL CBF image [14, 16]. First, R1 maps are derived from the Look-Locker multi-inversion time scheme, representing the longitudinal relaxation rate of the brain tissue. It has contrast similar to a T1-weighted image and, therefore, carries relatively detailed anatomical information. Arterial blood volume (aBV) maps are similar to low-resolution angiography maps, whereas arterial transit time (ATT) maps show the time necessary for the labelled blood to flow from the labelling slab to the vascular compartment of the imaging voxel. ATT maps can be useful to demonstrate the regions of prolonged transit time such as in steno-occlusive diseases [17]. In the normal brain, boundaries between the territories of the anterior, middle, and posterior cerebral arteries (watershed areas) have longer ATT than the core of these perfusion territories, thereby delineating areas prone to borderzone or watershed stroke [17].

This study introduces a visual QC system for the clinical evaluation of ASL perfusion maps. This visual QC system consists of two components: (1) a contrast component that indicates the image contrast between anatomical structures; and (2) an artifact component that scores the presence of image artifacts that degrade image quality, as previously proposed [1]. For a wide range of applicability, this visual QC system was not only developed for CBF maps, but also for other ancillary images that can potentially be acquired. The visual QC was evaluated in patients with a range of diseases, as well as in healthy volunteers.

## Methods

### Participants and study design

Data for this study originated from several previous studies regarding stroke, multiple sclerosis, QUASAR reproducibility [9, 18–21] as well as brain involvement in human immunodeficiency virus (HIV) infection and ECST-2 (Second European Carotid Surgery Trial, http://s489637516.websitehome.co.uk/ECST2/index2.htm); these latter performed at University College of London Hospitals (UCLH) and never published. QUASAR ASL scans of 158 subjects (age 47 ± 17.6, mean ± standard deviation; range of 18–98 years; 81 males) from six Philips 3T scanners from five different centres were included. The mixed study population consisted of 60 healthy volunteers (age 33.7 ± 8.9, mean ± standard deviation; range of 18–65 years; 34 males) from the QUASAR reproducibility study [1, 18, 20], 48 multiple sclerosis patients, 41 stroke patients and nine HIV-positive subjects. All studies were ethically approved by the Research Ethics Committees of studies used in this retrospective analysis (the multiple sclerosis patient study was approved by the Central London Research Ethics Committee number 09/H0715/45; the European Carotid Surgery Trial 2 (ECST2) by the National Research Ethics Service Committee – East of England, ref.: 11/EE/0347; the HIV patient study by the South East Coast – Surrey Research Ethics Committee 12/LO/0073; the QUASAR Reproducibility Study by the Singapore National Healthcare Group’s Domain Specific Review Board DSRB-A-036). Written informed consent was provided by all subjects.

### QUASAR image acquisition and processing

All imaging was performed on Philips 3T scanners (Achieva, Philips Healthcare, Best, The Netherlands) using the following QUASAR pulse sequence parameters: repetition time / echo time = 4000/22.5 ms; 13 inversion times between 40 ms and 3640 ms with an interval of 300 ms, flip angle 35°; field-of-view 240 × 240 mm^2^; matrix 64 × 64; seven slices of 6-mm thickness with a 2-mm gap, resulting in a 3.75 × 3.75 × 8 mm^3^ resolution. Label slab thickness was 150 mm; label gap 15 mm; vascular crushers set at 3 cm/s. All data were processed with QUASAR software [9, 16] written in Interactive Data Language (IDL 8.2, ITT Visual Information Solutions, Boulder, CO). Image processing and quantification were performed according to recent consensus [1]. All images were evaluated in native ASL space using ImageJ (National Institutes of Health, Bethesda, MD, USA, v. 1.52e) [22].

### Visual QC

The visual QC score composed of two parts. The contrast-based QC (cQC) described the visual contrast between anatomical structures and it can be used not only for CBF maps but also for the ancillary parametric maps (R1, aBV, ATT). The scores had a value between 0 and 2, with three items for each cQC maps, with a maximal value of 6 per map (i.e. CBF, R1, aBV, and ATT maps), totalling into 24 for these four cQC maps. The artifact-based QC (aQC) evaluated image quality with respect to common artifacts that can affect ASL CBF maps, and was only used for the CBF maps. Each of the four aQC items (motion, signal drop, distortion, and bright spots, as described below) had a value between 0 and 2, totalling a max of 8. The total QC score then had a maximum value of 24 + 8 = 32 (Table 1).

**Table 1:** Items evaluated for the visual quality control of ASL images

Evaluation of CBF maps	
Contrast component	
Grey matter (0–2)	
Grey/white matter differentiation (0–2)	
Basal ganglia and thalami (0–2)	
Subtotal (0–6)	
Artifact component	
Motion (0–2)	
Signal drop (0–2)	
Distortion (0–2)	
Bright spots and areas (0–2)	
Subtotal (0–8)	
Grand total (0–14)	
Evaluation of ancillary maps (QUASAR-specific)	
Contrast	
R1	
Grey matter (0–2)	
Grey/white matter differentiation (0–2)	
Basal ganglia and thalami (0–2)	
Subtotal (0–6)	
aBV	
Anterior cerebral arteries (0–2)	
Middle cerebral arteries (0–2)	
Posterior cerebral arteries (0–2)	
Subtotal (0–6)	
ATT	
Anterior watershed area (2)	
Posterior watershed area (2)	
Deep watershed area (2)	
Subtotal (0–6)	
Grand total (0–18)	

A higher score means a better contrast or less artifacts. Range of scores within parentheses. *ASL* arterial spin labeling, *CBF* cerebral blood flow, QUASAR quantitative signal targeting with alternating radiofrequency labelling of arterial regions, *R1* longitudinal relaxation rate, *aBV* arterial blood volume, *ATT* arterial transit time

### Contrast-based QC

For each image, the contrast visibility of three items was assessed (Table 1). Each item was scored from 0 to 2, as follows: clearly visible contrast (score 2), unclear contrast (score 1) or no visible contrast (score 0). The total score of each map (CBF, R1, aBV, ATT) had a maximum value of 6, for a maximum achievable cQC score value of 24. Higher scores equate to higher image contrast.

**Fig. 1:**
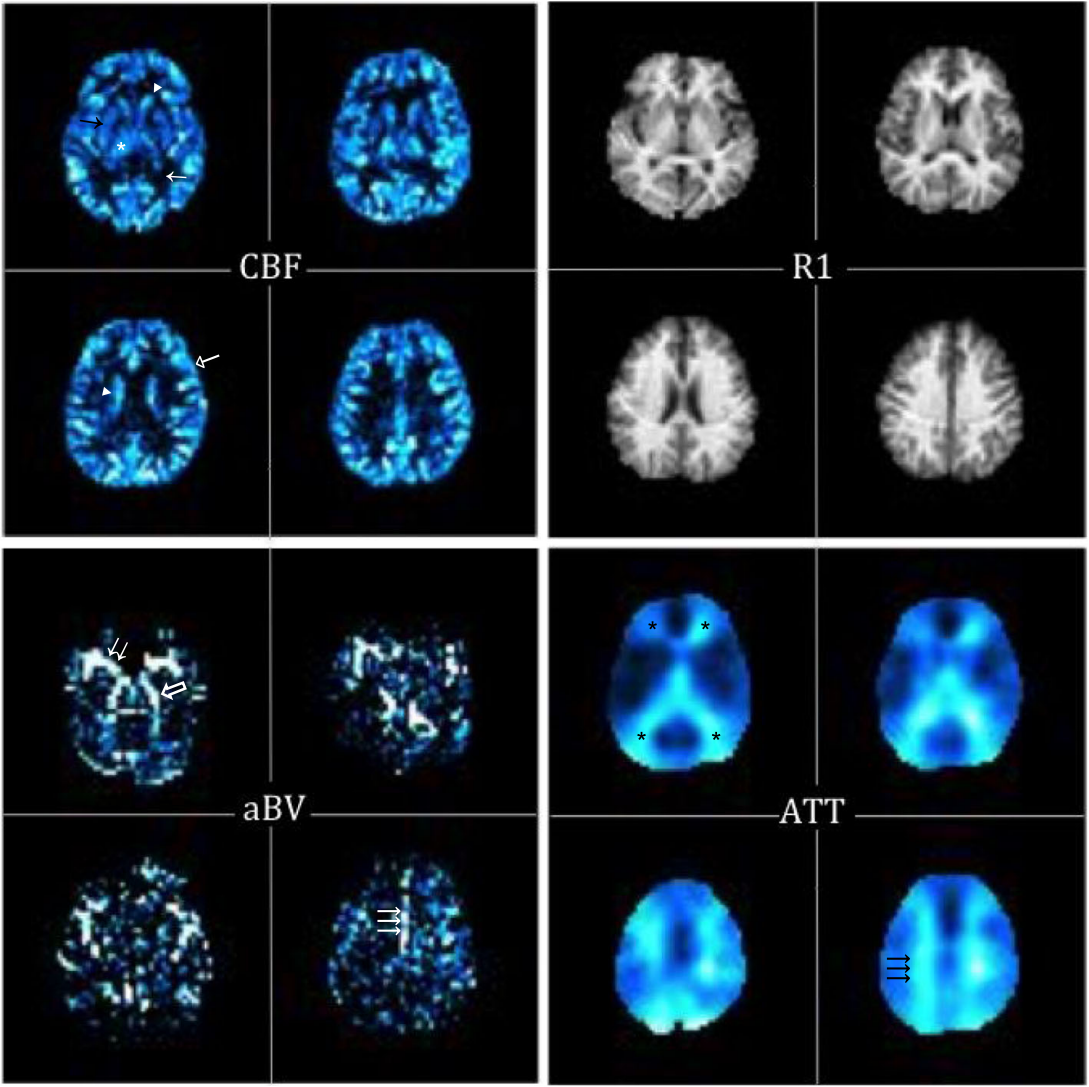
The cerebral blood flow (CBF) and T1 relaxation rate (R1) maps in the top row show an example of good contrast between the white matter and the cortical and subcortical grey matter: caudate nucleus head and body (arrowheads), thalamus (asterisk), white matter (arrow), grey matter (open-headed arrow). The arterial blood volume (aBV) maps illustrate the larger arterial volume corresponding to the anterior (triple arrow), middle (double arrow) and posterior cerebral (open arrow) arteries. The arterial transit time (ATT) maps show the areas of prolonged ATT in the superficial (asterisks) and deep (triple arrows) watershed areas

For the CBF and R1 maps, the three cQC items were the cortical grey matter, deep grey matter (i.e. basal ganglia and thalami), and grey matter (GM) to white matter (WM) differentiation. For the aBV maps, these were the contrast visibility of the three major intracerebral arteries: bilateral anterior, middle, and posterior cerebral arteries. These arteries appear as high-intensity vessels on the aBV maps. In the case of low scores, the anatomical images were reviewed to exclude arterial occlusion. For the ATT maps, the three cQC items investigated were the anterior and posterior superficial watershed areas, and the deep watershed area, which lie at the borders of major arterial territories [23]. These watershed areas were evaluated as prolonged ATT times on the ATT maps (Fig. 1).

### Artifact-based QC

**Fig. 2:**
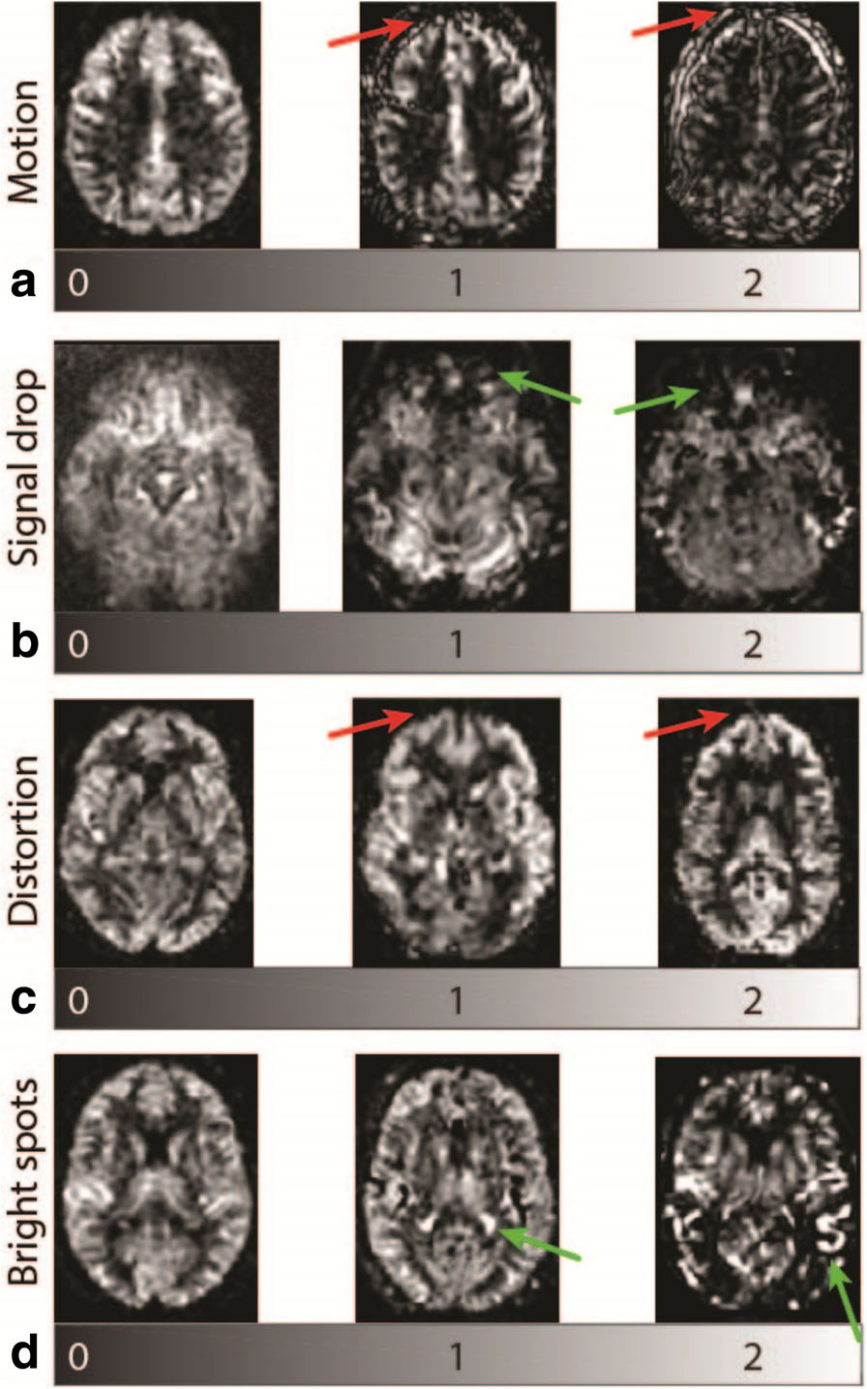
Cerebral blood flow images containing examples of artifacts: **a** motion artifacts are observed as pseudo-perfusion hyperintensity rings (red arrows), **b** signal drop in the inferior orbitofrontal region (green arrows), **c** geometric anterior-posterior distortion (red arrows), **d** bright spots depicting macro-vascular artifacts from the posterior cerebral artery behind the thalamus (1) and in the cortex (2, green arrows). Note that the 0 signal drop image is a 3D spiral image, for a better contrast on how the inferior orbitofrontal perfusion should appear without signal drop.

Four types of artifacts were assessed on the CBF maps: head motion, signal drop, geometric distortion, and macro-vascular bright spots (Fig. 2). Each item was scored from 0 to 2: no artifacts (score 2), moderate artifacts (score 1) or severe artifacts (score 0). The maximum achievable aQC score was 8, with higher scores equating to fewer image artifacts. Motion artifacts were detected as a hyperintensity rim around the CBF maps (Fig. 2a), which are due to the subtractive nature of ASL.

Signal drop (Fig. 2b) and geometric distortion (Fig. 2c) are the consequence of echo-planar imaging magnetic susceptibility at brain tissue-bone-air interface (susceptibility artifacts). Signal drop occurs frequently in the medial temporal cortex near the mastoid air cells at the base of the skull, as well as in the orbitofrontal cortex near the paranasal sinuses [24]. Some signal drop at the base of the skull is inevitable, and this was only scored when excessive aeration of the sinuses or petrous bone – defined as hyperpneumatisation – degraded the image contrast. Geometric distortion was defined as alterations of the outer contour of the image.

Macro-vascular artifacts are recognised as bright spots, due to voxels with a large aBV containing residual labelled blood in the large vessels. A typical example of a macro-vascular artifact of the middle cerebral artery is shown on the right in Fig. 2d. Macro-vascular artifacts or bright spots were defined as irregular, asymmetrical, vessel-shaped, high-intensity clusters, combined by a surrounding or distal, low-intensity area. Visual or auditory activation can mimic bright foci/spots in the primary visual and auditory cortices [24]. However, these are more often observed as a larger homogeneous area, often bilateral, and not accompanied by a surrounding or distal, low-intensity region [25]. Noise and motion may also present as bright spots [24, 25]. Care was taken to differentiate these causes of bright spots, by the above radiological image features as well as by the knowledge of vascular anatomy, although the latter can differ between subjects.

### Raters

All maps were independently evaluated within the same time period by three neuroradiologists, S.F. F.P. and B.G. with, respectively 7, 10, and 17 years of experience. Before rating, the raters had a training session to agree on how to score image contrast and artifacts. S.F. performed the rating of all data twice with an interval of 2 months, to assess the intra-rater agreement. Two raters, F.P. and S.B. independently performed an evaluation of the CBF maps to determine whether these were clinically usable or not. A senior neuroradiologist (28 years of experience), R.J. revised any disagreement and provided the final decision as to whether the scans were clinically usable or not. This binary evaluation was used as a reference to define a ‘clinically valid’ threshold using the receiver operating characteristic (ROC) analysis.

The scoring performed by the rater F.P. was only included in the agreement analysis, being excluded from the ROC analysis because she had participated in the binary classification that was used as a reference.

### Statistical analysis

The Spearman correlation coefficient (*r*) was used to investigate the relationship between image contrast and artifact scores (cQC and aQC). Intraclass correlation coefficients (ICC) were calculated to determine the levels of inter- and intra-rater agreements. ICC values were interpreted according to the following categorisation: 0 ≦ unusable < 0.2 ≦ poor < 0.4 ≦ fair < 0.6 ≦ good < 0.8 ≦ excellent ≦ 1.0 agreement [26, 27].

CBF, cQS, aQS, and total visual QC ROC curves were plotted with different thresholds to assess their performance in differentiating clinically usable and unusable ASL scans. Optimal thresholds were defined as those resulting in the maximum area under the curve (AUC). Sensitivity, specificity, positive and negative predictive values for differentiating between usable and non-usable ASL scans were also calculated.

## Results

**Fig. 3:**
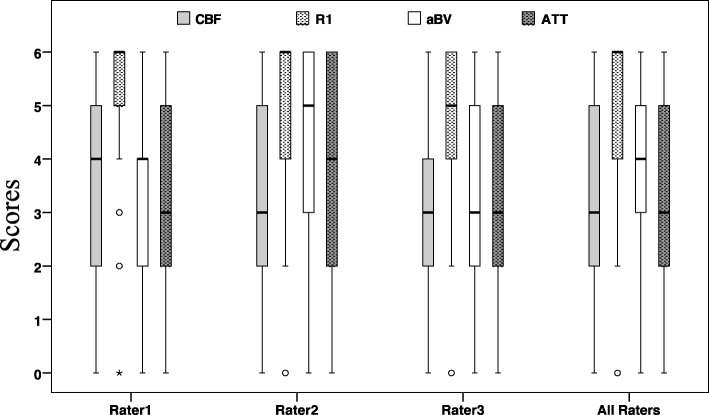
Visibility scores for each of the individual raters and all the data from the three raters. The T1 relaxation rate (R1) map has the highest score, median score of 6 averaged for all raters

Boxplots in Fig. 3 illustrate the QC scores of the three raters individually for each of the maps. R1 maps consistently showed the highest image contrast (median = 6). The CBF cQC and total QC scores correlated strongly with the aQC (*r* = 0.75, *p* < 0.001 and *r* = 0.70, *p* < 0.001, respectively, Table 2). Scans with poor cQC on the aBV maps also scored low on the CBF and ATT maps.

**Table 2:** Spearman’s correlation coefficient between the first (QC) and second (items) columns for raters individually (columns 3,4 and 5) and for all raters in the last column

QC	Item	Rater 1	Rater 2	Rater 3	All
cQC	CBF	0.85	0.78	0.60	0.75
cQC	R1	0.59	0.46	0.56	0.48
cQC	aBV	0.57	0.40	0.36	0.42
cQC	ATT	0.48	0.56	0.36	0.46
QC	Total cQC	0.80	0.74	0.60	0.70
aQC	Motion	0.70	0.56	0.65	0.62
aQC	Signal dropout	0.24	0.28	0.44	0.29
aQC	Distortion	0.21	0.25	0.22	0.22
*aQC*	Bright spots	0.58	0.63	0.26	0.48

*aBV* arterial blood volume *ATT* arterial transit time, *CBF* cerebral blood flow, *aQC* artifact-based quality control, *cQC* contrast-based quality control, *R1* T1 relaxation rate

Whereas the total cQC correlated strongly with motion artifacts (*r* = 0.62, *p* < 0.001), it correlated weakly with signal drop and geometric distortion artifacts (*r* = 0.29, *p* = 0.001 and *r* = 0.22, *p* = 0.002), respectively. The macro-vascular bright spots artifacts correlated moderately with the cQC (*r* = 0.48, *p* < 0.001).

### Intra- and inter-rater agreement

The neuroradiologists agreed in 123 maps and disagreed in 35 maps. The intra-rater agreement was high for cQC (ICC = 0.90), for aQC (ICC = 0.80), and for the combined QC (ICC = 0.90, Table 3). The inter-rater ICC was good for cQC (ICC = 0.72), for aQC (ICC = 0.60), and for the combined QC (ICC = 0.74).

**Table 3:** Intra- and inter-rater intraclass correlation coefficient(ICC) values

ICC	CBF	R1	aBV	ATT	cQC	aQC	Total QC
Intra-rater	0.87	0.84	0.77	0.86	0.90	0.80	0.90
Inter-rater	0.70	0.50	0.45	0.63	0.72	0.60	0.74

*aBV* arterial blood volume, *aQC* artifact-based quality control, *ATT* arterial transit time, *CBF* cerebral blood flow, *cQC* contrast-based quality control, *R1* T1 relaxation rate

**Fig. 4:**
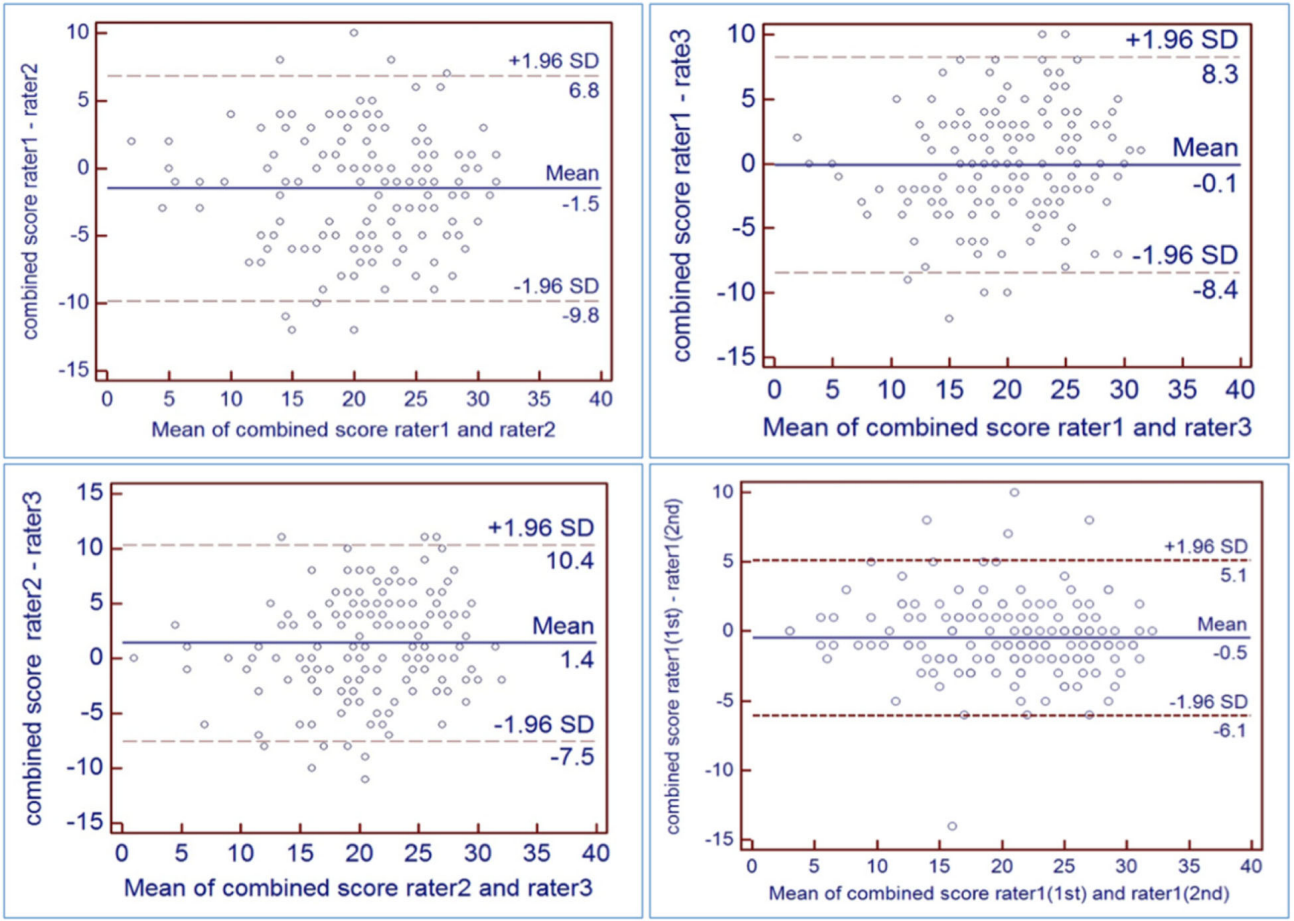
Bland-Altman plots illustrate inter (1–3) and intra-rater (4) variation of the combined score measurements. Red dashed lines are 95% CI of limits of repeatability, and the blue line is the mean difference.

Figure 4 shows intra- and inter-rater Bland-Altman plots for the combined QC score. Intra-rater, the 95% limits of agreement were ± 5.5 points, which equates to a within-subject coefficient of variation of 29.0% for a mean score of 19 points. While the mean difference between raters was less than 1.5 points in all three comparisons, the 95% limits of agreement for inter-rater variation (Fig. 4a, b, and c) were ± 8 points in all cases. This equates to a within-subject coefficient of variation of 42.1% for a mean score of 19 points.

### Diagnostic quality

Table 4 shows the sensitivity and specificity for the detection of clinically usable and non-usable ASL scans, using the cQC for the CBF images only. They were: 79 and 93%, respectively, for the CBF cQC at threshold value of 4/6; 85 and 80% for the total cQC at threshold value of 15/24; 87 and 76% for aQC at threshold value of 4/8; 90 and 80% for the total QC for threshold value of 18/32 (Table 4).

**Table 4:** Performance of CBF, QC, cQC, and aQC

	CBF cQC	Total QC	Total cQC	aQC
Score	4/6	19/32	15/24	4/8
Sensitivity (%)	75	87	88	90
Specificity (%)	90	73	73	68
PPV (%)	91	84	83	81
NPV (%)	70	78	79	82

*aQC* artifact-based quality control, *cQC* contrast-based quality control, *CBF* cerebral blood flow, *NPV* negative predictive value, *PPV* positive predictive value, *QC* quality control

**Fig. 5:**
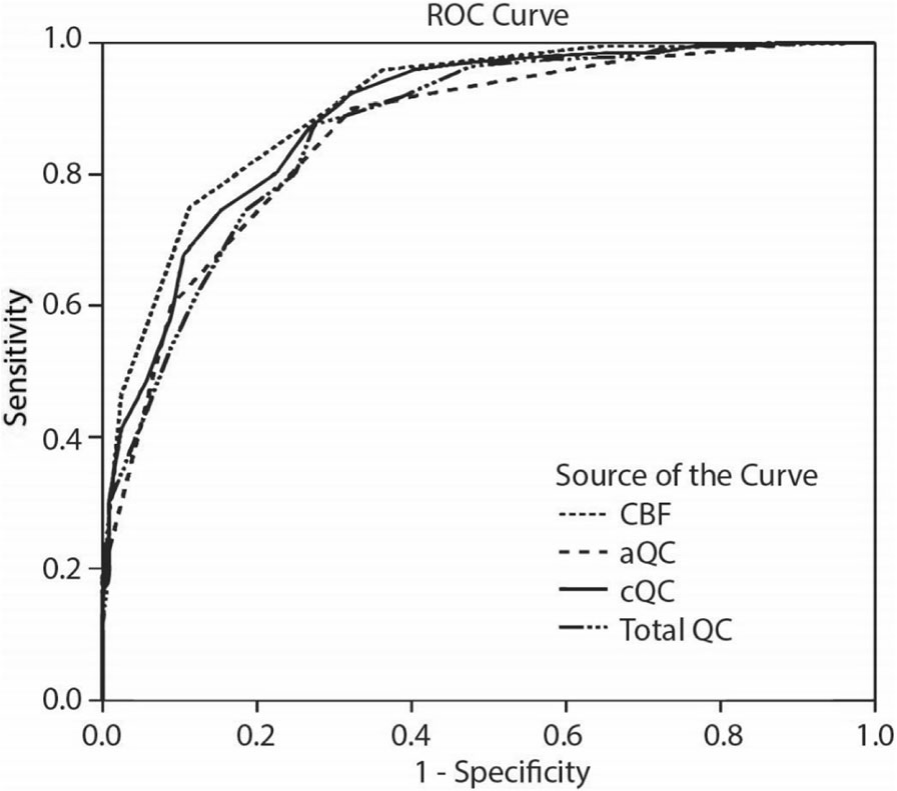
Receiver-operator characteristic (ROC) curves of cerebral blood flow (CBF) cQC, contrast-based quality control (cQC), artifact-based QC (aQC) and total QC. The areas under the curve for these 4 parameters are, respectively: 0.90, 0.89, 0.87, and 0.86

Whereas the CBF cQC had the highest AUC, 0.91 (0.88–0.94 95% confidence interval (CI)), the aQC had the lowest AUC, 0.88 (0.82–0.90 95% CI). AUC for the total cQC was 0.89 (0.83–0.91 95% CI) and 0.90 for the total QC (0.86–0.93 95% CI) (Fig. 5).

## Discussion

The main finding of this study is that the developed visual QC score system showed robustness within and between raters. The neuroradiologists felt this to be a helpful and easy-to-use rating system that provides an image quality indication before using ASL for any clinical or research assessment.

We provide several threshold scores as guidelines to determine whether or not the ASL image is of diagnostic quality. For the combined QC as used for QUASAR, a threshold of 18 seemed to be a robust choice. The fact that the CBF cQC score had a slightly higher performance can be explained by the fact that the reference for diagnostic usability was derived mainly from the CBF maps. Hence, for clinical single post-labelling delay pseudo-continuous ASL sequences, a CBF cQC threshold of 4 can be used as a diagnostic quality guideline. A CBF cQC score of 4 had the highest AUC (90%), and this threshold predicted clinical usability with specificity and sensitivity of 90 and 75%, respectively.

The high correlation between cQC and aQC may not be surprising, as image artifacts can obscure perfusion contrast. Interestingly, the motion aQC had the highest correlation with the cQC. This fits with a previous simulation study [28], which showed that motion had a smoothing effect on the contrast between GM and WM CBF across the brain. Motion artifacts are known to have a high impact on ASL image quality, due to the subtractive nature of the technique [1]. This is particularly emphasised for ASL sequences without background suppression, as was the case for QUASAR in this study.

The lack of a correlation between signal drop aQC and cQC could be explained by the fact that this susceptibility artifact is inevitable in several clinical magnetic resonance imaging (MRI) acquisitions, and are well-known and tolerated by radiologists. While its extent may vary largely between subjects because of difference normal variants of sinuses and air cavities, the locations and appearance of these artifacts are well-known. This led also to the pragmatic choice of accepting a slight signal dropout near the base of the skull as clinically usable, and normal. The other result of susceptibility artifacts − geometric distortion – also did not correlate with cQC. Geometric distortion is relatively mild in ASL compared to other advanced techniques, such as functional MRI and diffusion tensor imaging, as the readout length is typically shorter than in the latter techniques [29]. Moreover, this distortion does not necessarily change image contrast.

The raters had a good visual training regarding the main physiological and vascular anatomy variations expected in patients, so they did not report any particular difficulties to differentiate between them and the noise- or motion-related causes of bright spots. There was a moderate positive correlation between the bright spot aQC score and the general cQC (*r* = 0.48, *p* = 0.001).

Notably, although the primary goal of our study was to provide guidelines for radiologists to accept or discard an individual ASL scan based on visual QC, this scoring system can also be used as a teaching tool for neuroradiologists that are not familiar to ASL, to differentiate artifacts from perfusion changes.

The visual differentiation between normal anatomical variants and acquisition artifacts can be difficult. We acknowledge several conditions where pathology is known to simulate acquisition artifacts. A frequently occurring example is the signal drop due to a pathologically prolonged bolus arrival time. A more rare example is that the bright areas are explained by a pathological arteriovenous shunt [30]. In the first case, the typical anatomical vessel distribution of the signal drop areas can suggest the presence of an unknown extracranial vessel stenosis, while in the second one, the serpiginous shape of the bright areas, the bright signal in the main drainage veins and sinuses can indicate the presence of an arteriovenous shunt. The corresponding conventional anatomical MRI images can show a cluster of enlarged vessels in the latter case, due to an arteriovenous malformation, or a thin or absent flow void in T2- and T2*-weighted gradient-echo sequences of the intracranial portion of vessels, in case of a stenosis or occlusion. In doubtful cases, we suggest a pragmatic approach of taking into account the corresponding anatomical images and comparing them to the ASL maps to avoid treating physiological changes as image artifacts.

This study suffers from several potential limitations. Both the scoring system and the diagnostic usability which was used as a reference, are subjective. Future work may compare this clinical visual scoring system with existing parametric scoring systems, which may be more objective [31–34]. However, these parameters are sensitive to both instrumental and (patho-)physiological changes, making them less reliable for clinical use. This is especially important in the case of ASL, because its signal-to-noise ratio is directly related to CBF and other physiological alterations, such as haematocrit or oxygenation changes, hence clinical knowledge is required to distinguish instrumental artifacts from artifacts that are disease-related and inevitable [35]. Future work should investigate the combined performance of a visual and parametric QC in clinical applications of ASL.

On the other hand, the fact that image contrast and the presence of motion and vascular artifacts are disease-related in ASL, is a potential limitation to our visual QC as well. To this end, we included both ASL scans from patients and healthy controls. Although pathology could also affect our scoring, these changes are often focal compared to a more widespread acquisition-related quality decrease. Nevertheless, there remain cases where the differentiation between acquisition- or pathology-related quality decreases are difficult to assess, e.g. the GM-to-WM contrast loss in labelling asymmetry could appear the same as in a unilateral infarct. To differentiate these, we recommend to evaluate the anatomical MR images and perform a double comparison between them, as suggested above.

We found that the scan quality in patients appeared visually inferior to that of healthy controls, which was correlated to motion and a loss of image contrast, and vascular artifacts. Nevertheless, the majority of patient scans were considered clinically usable in the binary classification. However, it should be noticed that the poorer cQC and aQC scores for ASL scans from patients need to be accepted to a certain degree.

Another limitation to this study is that we only tested our scoring system in QUASAR scans with a two-dimensional (2D) echo-planar readout, which are not the type of scans recommended by the white paper [1]. Although image contrast and artifacts are expected to appear similar on other ASL sequences with a 2D echo-planar readout, they may differ with three-dimensional (3D) ASL readouts. The main difference is a lower effective spatial resolution for the 3D readouts that are used in ASL: 3D spiral and 3D gradient and spin-echo, mostly because of their wider acquisition point-spread function [36, 37]. These sequences still have a lower GM-to-WM contrast, less visibility and higher sensitivity for motion artifacts. Furthermore, these 3D sequences have a lower degree of geometric distortion and signal dropout, especially the 3D spiral sequence.

In conclusion, the proposed scoring system provides a robust visual quality control for QUASAR ASL images. The scoring system has demonstrated the ability to select clinically useful scans and shows reasonable reproducibility and reliability. Our results encourage future efforts to expand on our quality control guidelines for this growing technique.

## Abbreviations

2D: Two-dimensional
3D: Three-dimensional
aBV: Arterial blood volume
aQC: Artifact-based quality control
ASL: arterial spin labeling
ATT: Arterial transit time
AUC: Area under the curve
CBF: Cerebral blood flow
cQC: Contrast-based quality control
GM: Grey matter
HIV: Human immunodeficiency virus
ICC: Intraclass correlation coefficients
QC: Quality control
QUASAR: Quantitative signal targeting with alternating radiofrequency labelling of arterial regions
R1: T1 relaxation rate
ROC: Receiver operating characteristic
WM: White matter
